# Generation of *in vivo* neural stem cells using partially reprogrammed cells defective in *in vitro* differentiation potential

**DOI:** 10.18632/oncotarget.14861

**Published:** 2017-01-27

**Authors:** Jong Soo Kim, Yean Ju Hong, Hyun Woo Choi, Hyuk Song, Sung June Byun, Jeong Tae Do

**Affiliations:** ^1^ Department of Stem Cell and Regenerative Biotechnology, College of Animal Bioscience and Technology, Konkuk University, Seoul, Republic of Korea; ^2^ Animal Biotechnology Division, National Institute of Animal Science, Rural Development Administration, Suwon, Republic of Korea

**Keywords:** teratoma, pluripotent stem cells, iPSCs, neural stem cells, differentiation

## Abstract

Pluripotent stem cells can be easily differentiated *in vitro* into a certain lineage through embryoid body formation. Recently, however, we reported partially reprogrammed cells showing some pluripotent characteristics, which failed to differentiate *in vitro*. Here, we attempted to generate neural stem cells (NSCs) from partially reprogrammed cells using an *in vivo* differentiation system involving teratoma formation. Partially reprogrammed cells formed teratomas after injection into immunocompromised mice, and NSCs could be isolated from these teratomas. These *in vivo* NSCs expressed NSC markers and terminally differentiated into neurons and glial cells. Moreover, these NSCs exhibited molecular profiles very similar to those of brain-derived NSCs. These results suggest that partially reprogrammed cells defective in *in vitro* differentiation ability can differentiate into pure populations of NSCs through an *in vivo* system.

## INTRODUCTION

Pluripotent stem cells (PSCs) are able to differentiate into cells of all three germ layers [1, 2]. Differentiation of PSCs is easily induced *in vitro* in the absence of leukemia inhibitory factor (LIF) or other self-renewal supporting factors through embryoid body formation [3]. PSCs can also be differentiated *in vivo* through the formation of a chimera, in which PSCs recapitulate normal development [4, 5]. Interestingly, different types of PSCs show various levels of differentiation potential. Naïve PSCs can form chimeras, but primed PSCs lack the ability to form chimeras after blastocyst injection, although primed PSCs form chimeras after injection into embryos 7.5 days post coitum (dpc) [6, 7]. Recently, we generated a novel cell type, partially reprogrammed cells that show some pluripotent characteristics but are clearly distinguishable from fully reprogrammed iPSCs. They can form teratomas, which contribute mainly to the endoderm and ectoderm lineages, but are unable to differentiate in an *in vitro* culture system. These partially reprogrammed cells were not able to differentiate *in vitro* because they failed to form embryoid bodies [8]. Thus, to obtain differentiated cells from PSCs, we considered different differentiation protocols based on the types of PSCs.

The ability to form a teratoma is a characteristic of PSCs that distinguishes them from other cell types. Because a teratoma that forms from PSCs contains cell types of all three germ layers, teratomas can provide an *in vivo* differentiation environment that is a non-tissue-specific niche. Very recently, we developed an *in vivo* differentiation method by which neural stem cells (NSCs) can be derived from pluripotent embryonic stem cells (ESCs) through teratoma formation [9]. NSCs were isolated from cells of the teratoma tissue and established as stable cell lines. This method can be applied to differentiate PSCs into other cell types such as hematopoietic stem cells [10]. This report suggested that *in vivo* differentiation through teratoma formation is a powerful tool for differentiating PSCs into specific cell types. However, this *in vivo* differentiation method has yet to be tested with cells that are not fully pluripotent. Thus, in the present study, we examined whether this method for *in vivo* generation of NSCs through teratoma formation could be applied to partially reprogrammed cells that are defective in *in vitro* differentiation potential.

## RESULTS

### Embryoid body- and teratoma-forming ability of partially reprogrammed cells

**Figure 1:**
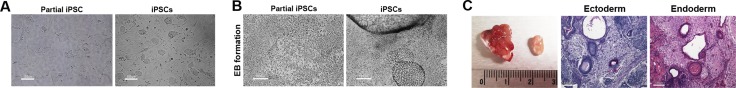
*In vitro* and *in vivo* differentiation potential of partially reprogrammed cells (**A**) Partial iPSCs formed flat colonies, whereas fully reprogrammed iPSCs formed dome-like colonies on feeder cell-layered dishes; scale bar = 100 μm. (**B**) Partial iPSCs did not form embryoid bodies (EB) using the *in vitro* differentiation protocol. In contrast, fully reprogrammed iPSCs successfully formed EBs; scale bar = 100 μm. (**C**) The *in vivo* differentiation potential of partial iPSCs determined by teratoma formation. Partial iPSCs formed teratomas, but mesodermal tissue was rarely detected. Teratoma tissue sections contained ectodermal and endodermal tissues; scale bar = 100 μm.

Recently, we generated partially reprogrammed cells, or partial iPSCs, that formed flat colonies without Oct4-GFP expression by transfection of a reprogramming factor-containing plasmid; the established cell line called XiPS-7 [8]. These XiPS-7 cells possess characteristics that clearly distinguished them from fully reprogrammed iPSCs. They formed relatively flat colonies exhibiting alkaline phosphatase activity and expressing Nanog, but not Oct4 [8]. Here, we confirmed the intermediate differentiation potential of the partially reprogrammed cells. The XiPS-7 cells formed flat colonies that were easily distinguished from the dome-like colonies from fully reprogrammed iPSCs (Figure 1A). When XiPS-7 cells were cultured for embryoid body formation in LIF-free medium, they were not able to form embryoid bodies and failed to differentiate *in vitro* (Figure 1B). Next, we determined the *in vivo* differentiation potential of XiPS-7 cells by analyzing teratoma formation. These partially reprogrammed cells were able to form teratomas after injection into the immunodeficient mice (Figure 1C). However, the teratoma tissues generated from partially reprogrammed cells mainly contained ectodermal and endodermal tissues, and rarely mesodermal tissue (Figure 1C). If the ectodermal tissues in the teratoma contained NSCs, these NSCs could be isolated and cultured *in vitro*.

### *In vivo* generation of NSCs from partially reprogrammed cells

Next, we explored the potential for *in vivo* generation of NSCs through teratoma formation using partially reprogrammed cells, which were not fully pluripotent. Because XiPS-7 cells do not contain the NSC-specific marker Olig2-GFP [9], putative NSCs could not be sorted by FACS. However, NSCs could be selected by culturing them in G418-containing NSC expansion medium. Host-derived cells and non-NSCs were eliminated in the selection medium; XiPS-7 cells were neo-resistant (carrying a *neo/lacZ* transgene), whereas non-NSC cells that were not resistant could not proliferate.

**Figure 2:**
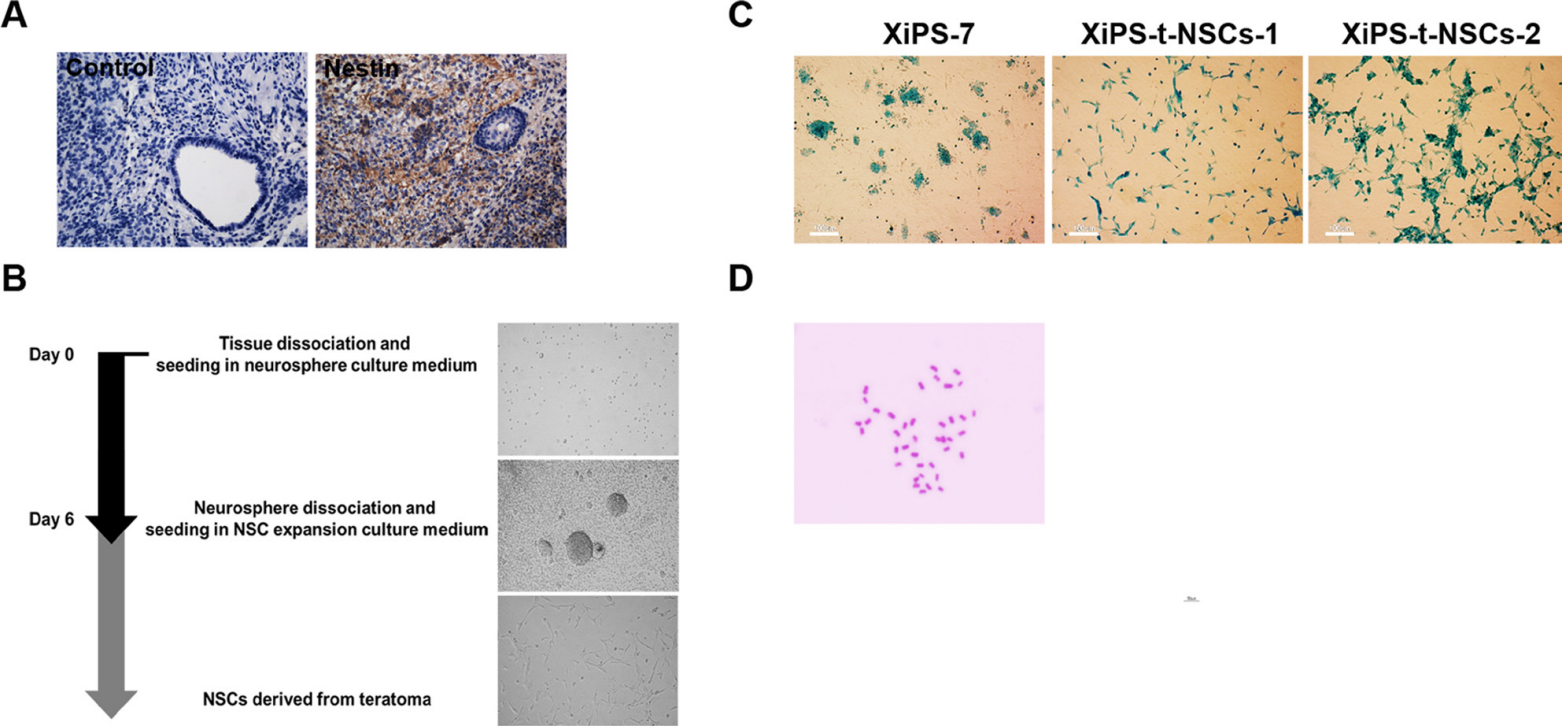
Successful generation of *in vivo* NSCs from teratomas derived from partially reprogrammed cells (**A**) Expression of Nestin in partial iPSC-derived teratoma sections; original magnification 100×. (**B**) Timeline of protocol for purification of NSCs from teratoma tissues. (**C**) Partial iPSCs and their teratoma-derived NSCs (XiPS-t-NSCs) were stained with X-gal, which indicated that there was no contamination by host cells; scale bar = 100 μm. (**D**) Karyotype analysis of XiPS-tNSCs. Forty diploid chromosomes were observed; DNA was stained with Giemsa.

**Figure 3:**
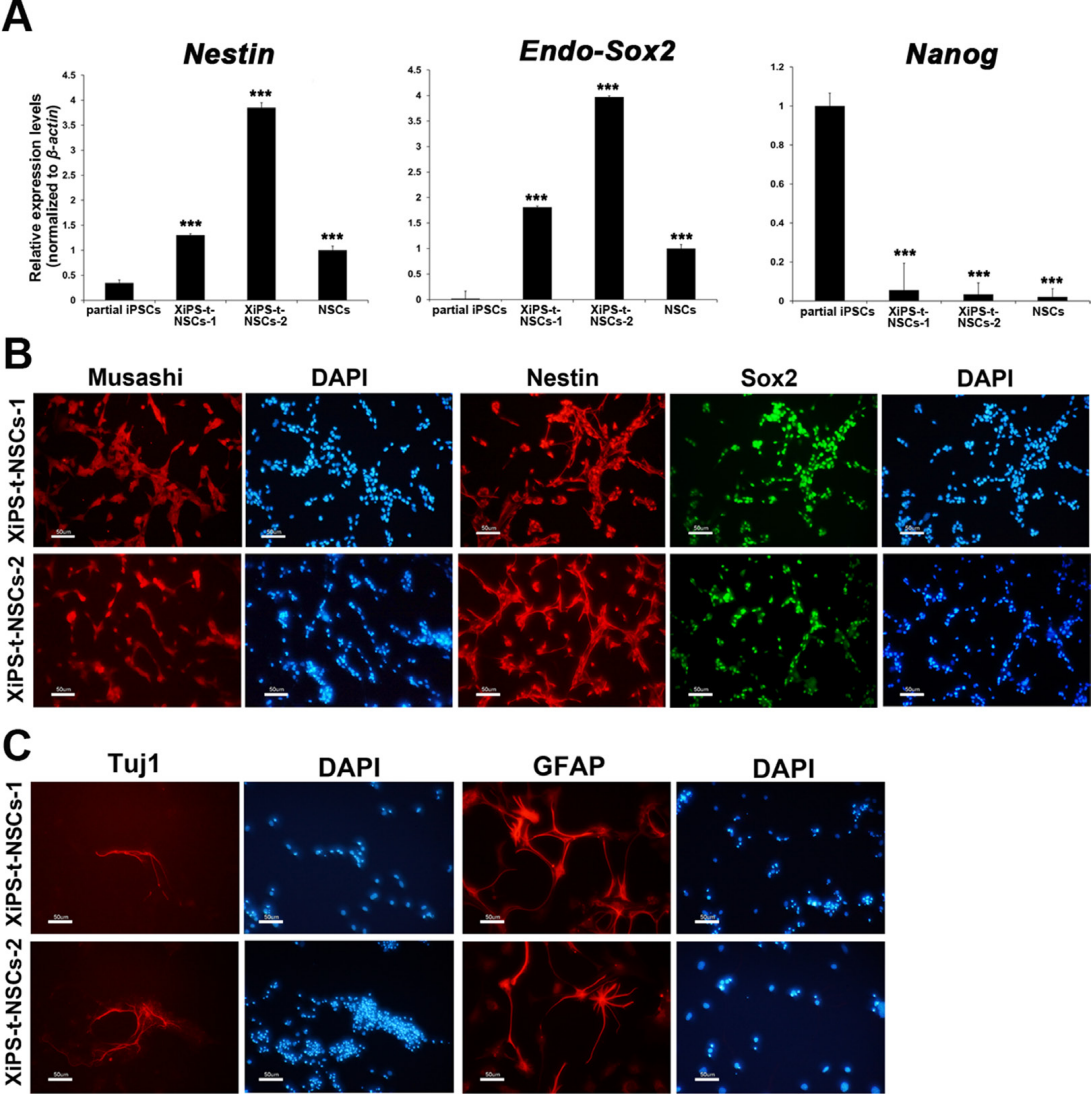
Characterization of *in vivo* NSCs from teratomas derived from partially reprogrammed cells (**A**) Real-time RT-PCR analysis for endo-Sox2 (endogenous), Nestin, and Nanog in partial iPSCs, XiPS-t-NSCs, and brain-derived NSCs. Relative expression levels were calculated by normalizing to β-actin; data represent mean ± SEM. Student’s *t*-test: ***p < 0.001 (**B**) Immunofluorescence staining of NSC markers Musashi, Nestin, and Sox2 in XiPS-t-NSCs, with DAPI counterstaining of nuclei; scale bar = 50 μm. (**C**) XiPS-t-NSCs differentiated into neurons (Tuj1+) and glial cells (GFAP+) *in vitro*. Nuclei were counterstained with DAPI; scale bar = 50 μm.

We obtained 4-week-old teratomas. As we found in the previous report, early-stage teratomas contained about 4 times more NSCs [9]. The NSC marker, Nestin, was detected in teratomas formed subsequent to the injection of XiPS-7 cells into the testis capsules of immunodeficient mice (Figure 2A). Dissociated single cells from 4-week-old teratomas were cultured in neurosphere medium, resulting in the death of most cells, with only a few cells forming neurosphere-like aggregates (Figure 2B). They also successfully formed neurospheres in non-adherent conditions (Supplementary Figure 1). Adherent NSCs (teratoma-derived NSCs from partial iPSCs; XiPS-t-NSCs, Figure 2B) formed following the transfer of neurosphere-like aggregates onto gelatin-coated dishes with NSC expansion medium. XiPS-t-NSCs were similar to brain-derived NSCs in morphology and were maintained over 20 passages (Figure 2B). To confirm that the XiPS-t-NSCs originated from the partially reprogrammed cells, the cells were stained with X-gal (Figure 2C); since the partially reprogrammed cells (XiPS-7) [8] were generated from OG2/ROSA26 *neo/lacZ* transgenic fibroblasts, their NSC derivatives were expected to stain with X-gal. As expected, all NSCs were stained by X-gal, indicating that there was no host cell contamination. Furthermore, these teratoma-derived NSCs contained 40 chromosomes, indicating that XiPS-t-NSCs were differentiated in teratoma, but nevertheless they maintain normal karyotype (Figure 2D). Real-time RT-PCR data showed that the NSCs derived *in vivo* expressed high levels of *Nestin* and endo-*Sox2*, but did not express *Nanog* (Figure 3A). Because the genomes of XiPS cells and XiPS-t-NSCs both contain exogenous *Sox2*, we selectively analyzed endogenous *Sox2*. Immunohistochemical analysis further demonstrated that these XiPS-t-NSCs were positive for the neural stem cell markers Musashi, Nestin, and Sox2 (Figure 3B). XiPS-t-NSCs could also differentiate into neuron and glial cell subtypes (Figure 3C and Supplementary Figure 2). These results suggest that partially reprogrammed cells that are defective in *in vitro* differentiation ability can differentiate *in vivo* and generate pure populations of NSCs through teratoma formation. The resulting XiPS-t-NSCs were indistinguishable from brain-derived NSCs in terms of morphology and expression of neural stem cell markers.

### Gene expression patterns of XiPS-t-NSCs are similar to those of brain-derived NSCs

**Figure 4:**
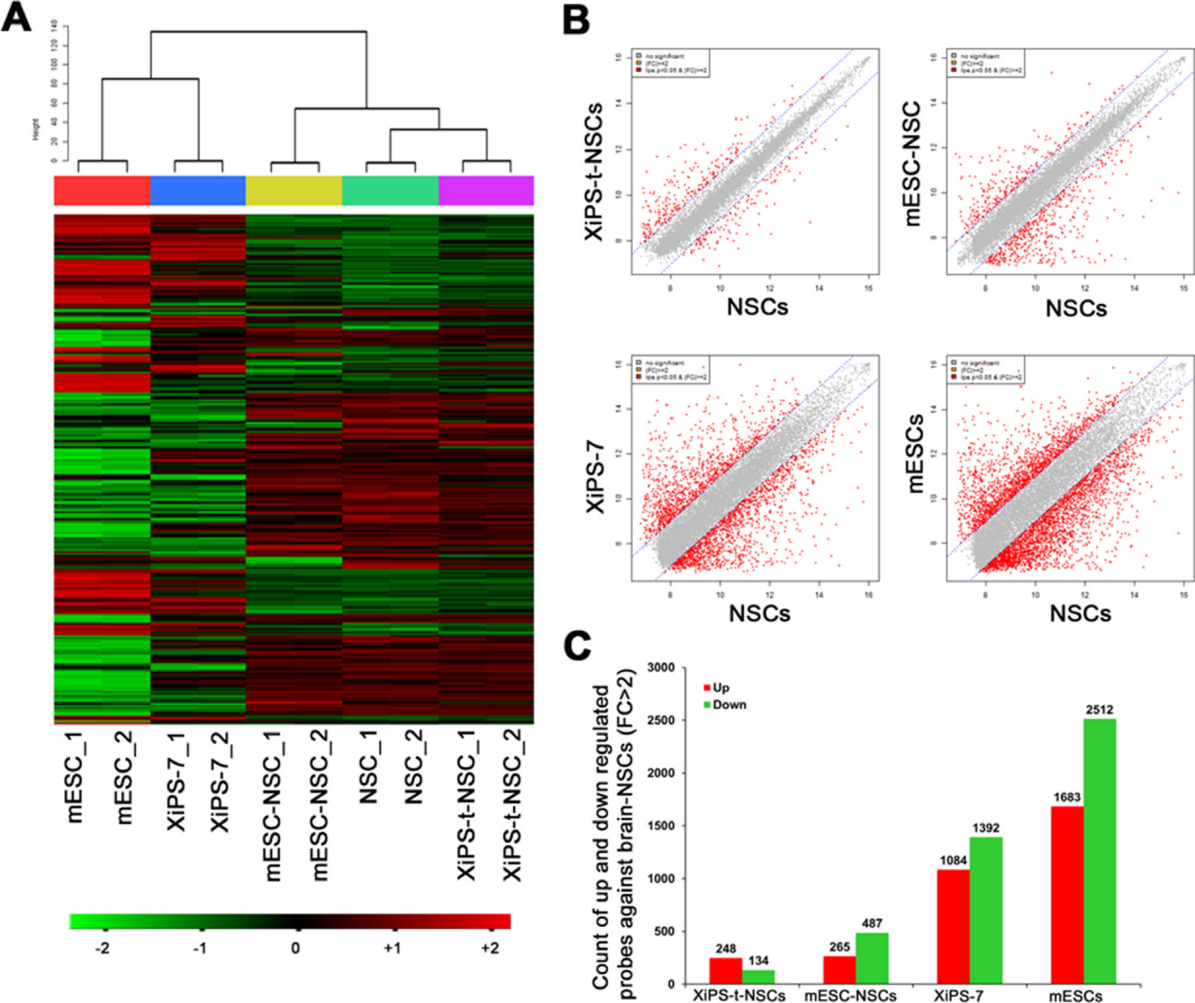
Comparative global transcriptome analyses of NSC lines (**A**) Hierarchical clustering of all genes using complete linkage and the Pearson distance analyses. (**B**) Scatter plot comparing XiPS-t-NSCs and NSCs, mESC-NSCs and NSCs, XiPS-7 and NSCs, and mESCs and NSCs. Gene expression levels are presented on a log2 scale. (**C**) Number of differentially expressed probes (fold change [FC] > ± 2 in comparison with that of NSCs. mESCs: Mouse embryonic stem cells; XiPS-7: Partially reprogrammed cells; mESC-NSCs: NSCs differentiated from mESCs (*in vitro*); XiPS-t-NSCs: NSCs differentiated from XiPS-7 by teratoma formation (*in vivo*); NSCs: 13.5 dpc embryo brain-derived NSCs.

We previously reported the global gene expression patterns of NSCs differentiated *in vitro* from two pluripotent cell types, EpiSCs (epiblast stem cells) and ESCs. Different from naïve pluripotent ESCs, EpiSCs are primed pluripotent state and derived from post-implantation epiblasts. NSCs differentiated from primed pluripotent EpiSCs are dissimilar to those of NSCs differentiated from naïve pluripotent ESCs [11]; the gene expression levels of ESC-NSCs were closer to those of brain-derived NSCs than those of EpiSC-derived NSCs. Thus, we examined the gene expression profiles of XiPS-t-NSCs, brain-derived NSCs, and mESC-derived NSCs (differentiated *in vitro*) by microarray analysis (Illumina’s MouseRef-8 v2 Expression BeadChip). Complete linkage and Pearson correlation analyses were used to cluster the cell types according to their global gene expression patterns. Heat map and hierarchical clustering analyses showed that the global gene expression pattern of XiPS-t-NSCs was more similar to that of brain-derived NSCs than to that of mESC-NSCs (Figure 4A). Scatter plot analysis and the number of up- and down-regulated probes (fold change [FC] > 2) showed similar results (Figure 4B and 4C). The number of up- and down-regulated probes for XiPS-t-NSCs vs. brain-NSCs was 248 and 134, respectively, which was less than the 265 up- and 487 down-regulated probes for mESC-NSCs.

## DISCUSSION

In this study, we found that *in vivo* differentiation through teratoma formation was a powerful tool for differentiation of cells that are not fully pluripotent into NSCs. NSCs differentiated in the *in vivo* system were more similar to brain-derived NSCs than those differentiated in an *in vitro* system from fully pluripotent ESCs. Thus, we suggest that this *in vivo* differentiation protocol may represent a universal method, because it can be applied to a variety of cell types, ranging from ESCs to partial iPSCs. In contrast, *in vitro* differentiation requires protocols that vary by cell type [12]. Moreover, the *in vivo* differentiation method has an advantage over *in vitro* differentiation in its efficiency in generating NSCs [9].

Recently, we developed two *in vivo* methods to differentiate PSCs into NSCs, each based on the teratoma- and chimera-forming abilities of PSCs [9], in press. Teratomas are not an NSC-specific niche, but it enables pluripotent cells to recapitulate NSC development. On the other hand, brains of chimeras can provide an appropriate niche for NSC development. Thus, brain tissue in a chimera seems to be a better environment for NSC generation than a teratoma. However, considering the formation of a type of mini brain tissue or neural rosette, it is likely that teratomas also provide a microenvironment for neurogenesis from PSCs or partially reprogrammed cells.

Other cell types such as hepatocytes and hematopoietic stem cells have been derived from ESCs and iPSCs [10, 13, 14]. Therefore, the *in vivo* differentiation approach presented here can contribute to the development of differentiation protocols for other cell types, for which *in vitro* systems have not been developed.

Previously, we reported that the gene expression profiles of NSCs differentiated from ESCs *in vitro* were more similar to those of brain-derived NSCs than those of EpiSC-derived NSCs [11]. These results suggest that naïve PSCs might recapitulate *in vivo* development better than primed PSCs. However, in the present study, we showed that even partial iPSCs, cells that were not fully pluripotent, could differentiate into NSCs that were more similar to brain-derived NSCs than ESC-NSCs (Figure 4). These results suggest that the differentiation environment is more important than the source of the cells that are subjected to differentiation.

Neurogenesis begins at an earlier stage of gastrulation during embryonic development [15]. This was also true in the *in vivo* differentiation system, because higher numbers of NSCs could be harvested from the earliest recoverable teratoma at 4 weeks post injection in immunocompromised mice [9]. This timeframe was similar to that for deriving NSCs from cells lacking selection markers, such as Olig2-GFP cells. We have not tried NSC differentiation through teratoma formation using cells that do not contain any selection markers such as Olig2-GFP or neo; however, it may be possible to obtain NSCs by culturing the rosette tissue from teratomas in NSC culture medium.

The findings of this study, in addition to those from our previous study, show that the *in vivo* differentiation protocol is applicable to many cell types, including cells that are not fully pluripotent, and NSCs very similar to brain-derived NSCs could be derived using this protocol. This approach can be further improved to provide cells for therapeutic applications using human ESCs or iPSCs.

## MATERIALS AND METHODS

### Cell culture of partially reprogrammed cells

Partial iPSCs (XiPS-7) were grown on mitomycin C-treated mouse embryo fibroblast (MEF) feeders with standard mouse ESC culture medium: Dulbecco’s modified Eagle’s medium (DMEM; Gibco) supplemented with 15% fetal bovine serum (FBS; Gibco), 1× penicillin/streptomycin/glutamine, 1 mM nonessential amino acids (NEAA; Gibco), 0.1 mM β-mercaptoethanol (Gibco), and 1000 U/mL leukemia inhibitory factor (LIF; ESGRO, Chemicon).

### Generation of partial iPSCs

We used mouse OG2/Rosa26 transgenic embryonic fibroblasts (MEFs) as somatic cells for reprogramming. OG2/Rosa26 heterozygous double transgenic MEFs were generated by crossing the Rosa26 (carrying the *neo/lacZ* transgene) strain with the OG2 transgenic strain (carrying green fluorescent protein [GFP] under the control of the *Oct4* promoter) [16, 17]. Partially reprogrammed cells were derived by transfection of a reprogramming factor-containing plasmid using Xfect^TM^ transfection reagent (Clontech) [8]. The plasmids pCX-OKS-2A (Oct4 [O], Klf4 [K], and Sox2 [S], each separated by a different 2A sequence] and pCX-cMyc were purchased from Addgene. The plasmids were mixed with 3 μg pCX-OKS-2A and 1 μg pCX-cMyc. MEFs were seeded at 1 × 10^5^ cells/well in 6-well plates (day 0). Plasmids were introduced using 1.2 μL of XfectTM transfection reagent (Clontech) according to the manufacturer’s instructions. From day 4, the transfected MEFs were cultured in mouse ESC culture medium containing LIF. On day 9, the cells were harvested with trypsin and plated on 100-mm dishes with MEF feeder cells. On days 25–28, Oct4-GFP-positive or -negative colonies were picked for expansion and maintained in mouse ESC culture medium. Among the mixed population of cultured cells, ESC-like cells that formed relatively flat colonies and did not express Oct4-GFP were picked and cultured under ESC conditions. These Oct4-GFP-negative ESC-like colonies were maintained up to 20 passages and were designated the XiPS-7 cell line.

### Teratoma formation

Immunodeficient Balb/c Nude (5-week-old, male) mice were purchased from Orient Bio (Gyeonggi-do, Korea) for use in experiments. Here, 1 × 10^6^ partial iPSCs were injected into the testis capsule of immunodeficient mice. Teratomas were harvested surgically from mice at 4 weeks post injection. Dissected teratomas were fixed in 4% paraformaldehyde (Sigma), processed through graded ethanol, and embedded in paraffin, followed by hematoxylin/eosin (endoderm) and periodic acid-Schiff (PAS, ectoderm) staining. Immunohistochemical staining of Nestin performed with an anti-Nestin antibody (Nestin; monoclonal, 1:100, Millipore).

### *In vivo* NSC derivation

Partial iPSC-derived teratomas were dissociated into single cells by 0.25% trypsin (Gibco) treatment. Cells were plated on suspension dishes containing NSC culture medium (DMEM/F12 (Gibco) supplemented with N2 supplement (Gibco), B27 supplement [(Gibco), 1× penicillin/streptomycin/glutamine (Gibco), 1 M HEPES (Gibco), 45% glucose (Sigma), 20 ng/mL EGF (Invitrogen), and 20 ng/mL bFGF (Invitrogen)], then cultured for 2 to 3 weeks. Neurospheres were then replated on gelatin-coated tissue culture dishes containing NSC expansion medium and cultured for 2–3 days. Expanded neurospheres were dissociated into single cells by 0.25% trypsin (Gibco) treatment and passaged every 2–3 days in NSC expansion medium.

### Differentiation of NSCs into neurons and glial cells

NSCs were differentiated into neurons and glial cells by 3 weeks of culture in neuronal differentiation medium (1:1 mixture of DMEM/F12 medium and neurobasal medium supplemented with 1% fetal bovine serum, N2 and B27 supplements, and 1× penicillin/streptomycin/glutamine [all Gibco]).

### Immunocytochemistry

NSCs were stained for NSC markers: Nestin, Sox2, and Musashi. For immunocytochemistry, cells were fixed with 4% paraformaldehyde (Sigma) for 20 min at 4°C. After washing with PBS (Gibco), cells were treated with PBS containing 3% bovine albumin serum and 0.03% Triton X-100 (Sigma) for 45 min at room temperature. The primary antibodies used were anti-Nestin (Nestin; monoclonal, 1:500, Millipore), anti-Sox2 (Sox2; polyclonal, 1:500, Millipore), and anti-Musashi-1 (Musashi-1; polyclonal, 1:500, Millipore). For detection of primary antibodies, fluorescently labeled (Alexa Fluor 488 or 568; Molecular Probes, Eugene, OR, USA) secondary antibodies were used according to the specifications of the manufacturer.

### RNA isolation and real-time quantitative real-time (qRT)-PCR analysis

Total RNA was isolated using an RNeasy Mini Kit (Qiagen, Venlo, Netherlands, http://www.qiagen.com) and then treated with DNase to remove genomic DNA contaminants. One microgram of total RNA was reverse-transcribed with a Super-Script III Reverse Transcriptase Kit (Invitrogen) and oligo (dT)_20_-primer (Invitrogen) according to the manufacturer’s instructions. The qRT-PCR reactions were performed in duplicate using a Power SYBR Green Master Mix (Takara) and Roche LightCycler 5480 [18]. The primers for these reactions were as follows: Nanog (endo) sense 5′-AGGCTGATTTGGTTGGTGTC-3′, Nanog (endo) anti-sense 5′-CCCAGGAAGACCCAC ACTCAT-3′; Nestin (endo) sense 5′-AAGTTCCCAGGCT TCTCTTG-3′, Nestin (endo) antisense 5′-GTCTCA AG GGTATTAGGCAAGG-3′; Sox2 (endo) sense 5′-CATGA GAGCAAGTACTGGCAAG-3′, and Sox2 (endo) anti-sense 5′-CCA ACG ATA TCA ACC TGC ATG G-3′.

### X-gal staining

Partial iPSCs or XiPS-t-NSCs, derived from OG2/Rosa26 transgenic mice, were stained with X-gal. Cells were rinsed with PBS and fixed in 4% paraformaldehyde for 20 min at 4°C. Cells were rinsed three times at room temperature in PBS containing 5 mM EGTA (Gibco), 0.01% deoxycholate, 0.02% NP40 (Sigma), and 2 mM MgCl_2_. Cells were then washed with PBS (Gibco) and stained in X-gal staining solution: PBS (Gibco) supplemented with 1 mg/mL 5-bromo-4-chloro-3-indolyl-galactosidase (X-gal; Promega), 5 mM K_2_Fe(CN)_6_, 5 mM K_4_Fe(CN)_6_, and 1 mM MgCl_2_. Blue staining was visualized by light microscopy.

### Microarray-based analysis

Total RNA was isolated using an RNeasy Mini Kit (Qiagen) and digested with DNase I (RNase-free DNase, Qiagen) according to the manufacturer’s instructions. Total RNA was amplified, biotinylated, and purified using an Ambion Illumina RNA amplification kit (Ambion) according to the manufacturer’s instructions. Labeled cRNA samples (750 ng) were hybridized to a MouseRef-8 v2 Expression BeadChip. Signals were detected using Amersham Fluorolink Streptavidin-Cy3 (GE Healthcare Bio-Sciences) according to the bead array manual. Arrays were scanned with an Illumina Bead Array Reader according to the manufacturer’s instructions.

Raw data were extracted using the software provided by the manufacturer (Illumina GenomeStudio v2011.1, Gene Expression Module v1.9.0). Array data were filtered with a detection *p*-value < 0.05 in at least 50% of the samples. Selected probe signals were log-transformed and normalized using the quantile method and then comparatively analyzed using a local-pooled-error (LPE) test and fold-change. The false discovery rate was controlled by adjusting the *p*-value with the Benjamini-Hochberg algorithm. Hierarchical clustering was performed using complete linkage and Pearson distance as measures of similarity.

### Statistical analysis

All experiments were performed in triplicate, and data represented as means ± SD. Differences were assessed by using the unpaired *t*-test and a *p*-value < 0.05 was considered significant.

## SUPPLEMENTARY MATERIALS FIGURES AND TABLES


