# Supplementary material for: Generation of in vivo neural stem cells using partially reprogrammed cells defective in in vitro differentiation potential
Source: Oncotarget. 2017 Jan 27;8(10):16456–62. doi: 10.18632/oncotarget.14861 (PMC5369976; doi:10.18632/oncotarget.14861)
Supplement: Supplementary file 1 [file oncotarget-08-16456-s001.pdf]

Generation of *in vivo* neural stem cells using partially reprogrammed cells defective in *in vitro* differentiation potential

Supplementary Materials

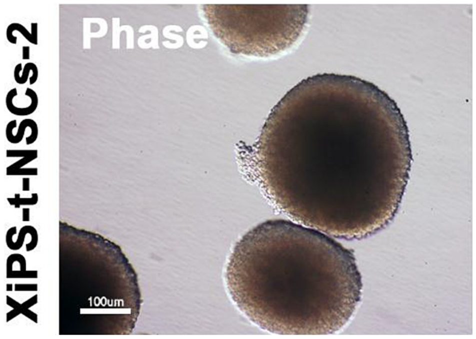

Supplementary Figure 1: Neurosphere formation of XiPS-tNSCs. Adherent XiPS-t-NSCs can form neurospheres on non-adherent condition, scale bar = 100 um.

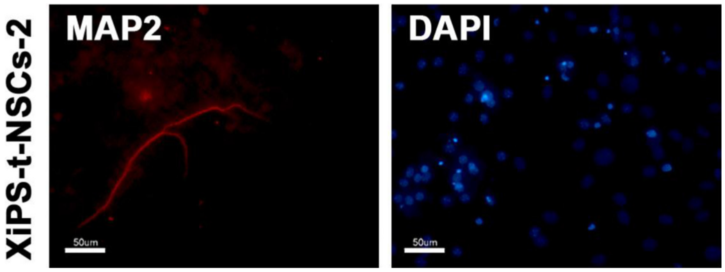

Supplementary Figure 2: Neuronal differentiation of XiPS-t-NSCs. XiPS-t-NSCs can differentiate into mature neurons (MAP2+) *in vitro*; nuclei were counterstained with DAPI; scale bar = 50 μm.
